# Insulin resistance surrogate markers and risk of hyperuricemia among patients with and without coronary artery disease: a cross-sectional study

**DOI:** 10.3389/fnut.2023.1048675

**Published:** 2023-08-21

**Authors:** Zeinab Ghorbani, Seyedeh Nooshan Mirmohammadali, Nargeskhatoon Shoaibinobarian, Sara K. Rosenkranz, Samira Arami, Azita Hekmatdoost, Marjan Mahdavi-Roshan

**Affiliations:** ^1^Cardiovascular Diseases Research Center, Department of Cardiology, Heshmat Hospital, School of Medicine, Guilan University of Medical Sciences, Rasht, Iran; ^2^Department of Clinical Nutrition, School of Medicine, Guilan University of Medical Sciences, Rasht, Iran; ^3^Department of Food, Nutrition, Dietetics and Health, Kansas State University, Manhattan, KS, United States; ^4^Department of Nutrition, School of Medical Sciences and Technologies, Islamic Azad University, Science and Research Branch, Tehran, Iran; ^5^Department of Kinesiology and Nutrition Sciences, University of Nevada, Las Vegas, Las Vegas, NV, United States; ^6^Department of Nutrition Research, National Nutrition and Food Technology Research Institute, Faculty of Nutrition and Food Technology, Shahid Beheshti University of Medical Sciences, Tehran, Iran; ^7^Department of Clinical Nutrition and Dietetics, National Nutrition and Food Technology Research Institute, Faculty of Nutrition and Food Technology, Shahid Beheshti University of Medical Sciences, Tehran, Iran

**Keywords:** hyperuricemia, insulin resistance, coronary artery disease (CAD), triglyceride, insulin sensitivity

## Abstract

**Background:**

Although emerging evidence emphasizes the associations between both insulin resistance and hyperuricemia with coronary artery disease (CAD) risk, no definite relationship has yet been established. In this respect, time-efficient and affordable methods to estimate insulin resistance (IR) status, and to predict risk of hyperuricemia, are needed. Thus, the goal of this investigation was to examine the associations between IR, as assessed by novel surrogate markers [triglyceride-glucose (TyG) and TyG–body mass index (TyG-BMI)], and risk of hyperuricemia in patients with and without diagnosed CAD.

**Methods:**

This cross-sectional study used data from the medical records of 1,170 patients who were referred to the cardiology outpatient clinic. Medical records, anthropometrics, and serum analytes were determined at the initial visit. Hyperuricemia was defined as serum uric acid ≥ 5.6 mg/dL. IR was estimated through surrogate markers (TyG and TyG-BMI). Multiple regression analysis was performed to assess the relationship between these indices and odds of hyperuricemia among patients with and without CAD.

**Results:**

Overall, 814 angiographically-confirmed CAD cases (mean age (SD) = 52 (8)yrs) were compared with 356 patients without CAD (mean age (SD) = 48 (8)yr). There were positive associations between TyG and TyG-BMI indices and odds of hyperuricemia in CAD patients after controlling for confounders (adjusted odds ratio (aOR) = 1.60; 95%CI: 1.02–2.51; *p*-value = 0.036; and aOR = 1.83; 95%CI: 1.24–2.70; *p*-value = 0.002, third tertiles for TYG and TYG-BMI, respectively).

**Conclusion:**

The present findings suggest that higher levels of the IR surrogate markers, TyG and TyG-BMI, are associated with higher odds of hyperuricemia in patients with CAD. However, given the cross-sectional design of this study, the sensitivity and specificity of these novel markers could not be determined for confirming the diagnosis of IR and hyperuricemia, further studies are needed to determine such outcomes and to confirm the current findings.

## Introduction

A high blood uric acid level is known as hyperuricemia. There have been several definitions proposed for diagnosing hyperuricemia, but from a clinical point of view, anything above the typical maximum limit of 6.8–7 mg/dL is regarded as saturated uric acid, and symptoms may appear accordingly that indicate the presence of gout (1). More recent evidence provided by research from the Uric Acid Right for heart Health (URRAH), a polycentric Italian cohort study investigating the threshold for serum uric acid (SUA) that is associated with the risk of cardiovascular disorders, suggested that SUA could independently predict risk for cardiovascular events and all-cause mortality when above 4.7–5.7 mg/dL (2–4). This elevated level is typically brought on by reduced uric acid excretion, elevated production of uric acid, or a combination of both factors (5). Approximately 21% of the general population and 25% of hospitalized patients have asymptomatic hyperuricemia (5), and it is far more common in men as compared with women (4,1 ratio) (6). This condition is not considered to be a health concern when patients are asymptomatic, though its most common and well-known complication is gout, which has a prevalence rate of approximately 3.9% in the U.S. population. Notably, hyperuricemia has become more prevalent in the past few decades, since it is frequently comorbid with obesity, metabolic diseases, type 2 diabetes mellitus (T2DM), dyslipidemia, hypertension, metabolic syndrome, chronic kidney disease, cardiovascular diseases, and cardiometabolic-related complications (5–7). The elevated occurrences of T2DM and metabolic syndrome, which frequently coexist with hyperuricemia, have emerged as prominent public health concerns. The interrelationships among these conditions have garnered scientific attention due to their potential implications for disease pathogenesis and management (7, 8). As reported in a meta-analysis of 11 cohort studies, T2DM risk is elevated by 17% for each 1 mg/dL elevation in SUA levels (9). Additionally, in accordance with the available evidence, people with metabolic syndrome may experience hyperuricemia due to insulin resistance, fatty liver, and dyslipidemia (7, 10).

Insulin resistance has been acknowledged as a general risk factor in many pathological conditions, including abnormal glucose tolerance, T2DM, metabolic syndrome, dyslipidemia, and obesity (11). Not only can insulin resistance begin up to two decades prior to the appearance of T2DM in non-diabetic patients, but it may also independently predict incident cardiovascular disorders (CVDs) and mortality (11). Impaired insulin sensitivity is thought to play an important role in the development of hyperinsulinemia and the progression of atherosclerotic-related conditions including hypertension, dysmetabolism, inflammation, endothelial dysfunction, and coronary artery disease (CAD), even among individuals without T2DM, or in the absence of any other clinical signs of insulin resistance. Although the underlying pathological mechanisms of these associations are not well-established, an accumulating body of research supports the potential for a causal role for reduced insulin sensitivity in increasing the risk of morbidity related to atherosclerosis, particularly for CAD and ischemic stroke (12–19).

Of note, in recent studies, the associations between insulin resistance as the principal symptom of T2DM and metabolic syndrome with elevated levels of SUA as emerging risk factors for CVDs have gained attention (7, 20, 21). Although emerging evidence emphasizes the link between both insulin resistance and hyperuricemia with CAD, no definite relationship has been established yet (7, 20). As such, it has been hypothesized that hyperinsulinemia might cause hyperuricemia, and that the reverse would not be true. Additionally, lowering serum urate levels is unlikely to improve insulin resistance and associated cardiometabolic consequences; conversely, alleviating insulin resistance may decrease serum urate levels and subsequent gout risk (22, 23).

Therefore, conducting a comparative investigation of patients with CAD and without CAD to assess the presence of insulin resistance and hyperuricemia could offer valuable insights into the potential associations between these risk factors and CVDs. Although the hyperinsulinemic-euglycemic clamp is considered the gold standard for determining insulin resistance, given the fact that it is invasive, time consuming, and expensive, conventionally in epidemiological studies and clinical practice, the Homeostatic Model Assessment for Insulin Resistance (HOMA-IR) has been recognized as a reliable and popular method to gauge insulin resistance, using fasting blood glucose and insulin (24, 25). However, there are also additional practical and feasible indirect methods that do not require serum insulin levels to approximate insulin resistance status. Therefore, predicting insulin resistance status through novel surrogate indicators, including the triglyceride glucose (TyG) and TyG-body mass index (BMI) indices, taking into account a combination of fasting triglycerides, glucose, and BMI status (as an indicator of obesity and excess body weight), may be more feasible and practical than the other costly methods (7, 26–29). TyG and TyG-BMI have been shown to accurately indicate lipotoxicity and glucotoxicity status (30, 31). Furthermore, similar to more well-known markers of insulin resistance, such as HOMA-IR, these novel biomarkers have also been shown to be significantly associated with CVD risk factors such as metabolic syndrome, arterial stiffness, diabetes, hypertension, coronary stenosis, and all-cause and/or cardiovascular (CV) mortality (30–36). In particular, Cho et al. conducted a cross sectional study that indicated that the TyG index was correlated with risk of obstructive CAD and CAD incidence, even following adjustment for traditional cardiovascular risk factors (25). Therefore, we sought to determine insulin resistance status using novel surrogate indices including TyG and TyG-BMI, along with SUA among patients with and without CAD, as these indices may serve as feasible and practical clinical assessments approximate insulin resistance, and to determine risk for hyperuricemia, and atherosclerotic-related conditions.

## Methods

### Participants

**Figure 1:**
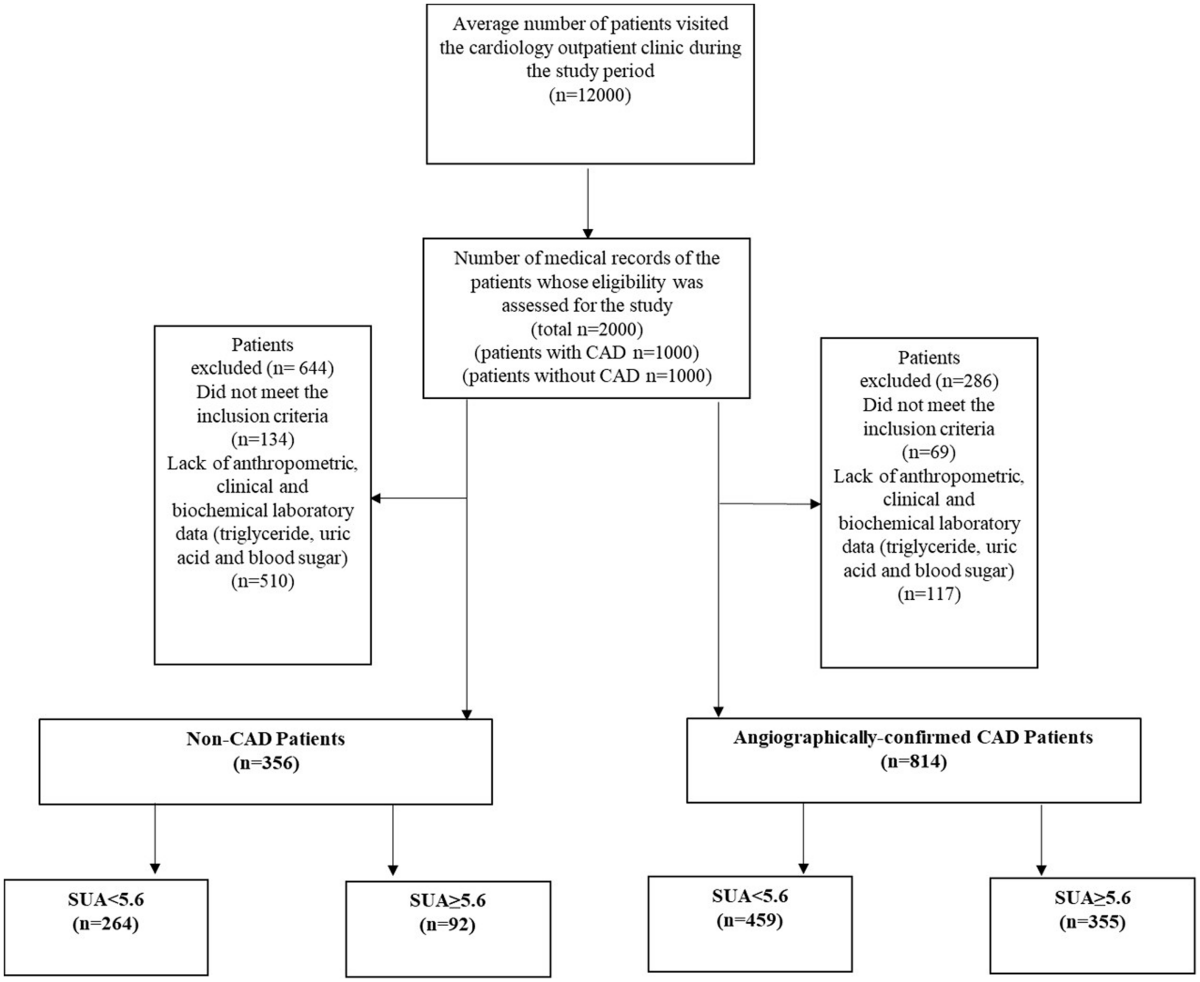
Participant flow diagram. CAD, coronary artery disease; SUA, serum uric acid.

In this single-center cross-sectional study, data were obtained from 2019 to 2021, when about 12,000 Iranian individuals visited the cardiology outpatient clinic at Dr. Heshmat Hospital in Rasht, Iran. Reasons for visiting the clinic varied from routine check-ups to having clinical signs of cardiac disorders. Expert cardiologists examined all patients to determine CAD diagnosis. Following reviews of a total of 12,000 medical records, those of 2000 patients were randomly selected for inclusion in the current study. Of the 2000 patients selected, cardiologists ruled out a CAD diagnosis in 1000 patients following the initial examination and assessment of clinical or laboratory signs of atherosclerotic conditions or angina pectoris. These assessments were also based on negative results for non-invasive tests (i.e., exercise stress tests, and/or echocardiography). For the remaining 1,000 patients, angiographic findings were then used to confirm CAD diagnosis in accordance with “*ESC 2019 guidelines for the diagnosis and management of chronic coronary syndromes*” (37). All patients who had the required demographic, medical history, and anthropometric data (such as weight and height) were considered for study inclusion. Patients with medical histories indicating a serious cardiac disorder, liver, kidney, neurologic diseases, cancer, thyroid dysfunction, gout according to physicians’ diagnosis or those who reported use of alcohol, vitamin C supplements, theophylline, or warfarin 3 months prior to study were excluded from the study. Accordingly, 644 subjects in the non-CAD group (134 did not meet the inclusion criteria, and 510 did not have anthropometric, clinical, and biochemical laboratory data past medical history, physician examination data, triglycerides, uric acid, and blood glucose), and 286 subjects in the CAD group (69 did not meet the inclusion criteria, and 117 lacked anthropometric, clinical, and biochemical laboratory data) were excluded from the analysis. In total, 1,170 patients (356 non-CAD and 814 CAD) between the ages of 30 and 75 years, with BMIs between 18.5 and 39 kg/m^2^ were included for analysis (Figure 1).

All research procedures were in line with the guidelines outlined in the 2013 version of the Declaration of Helsinki. The study protocol was assessed and approved by the Cardiovascular Diseases Research Center Institutional Review Board [affiliated with Guilan University of Medical Sciences (GUMS)] (registered with research number = 4,246). The GUMS’ Ethics Committee also approved this study (ethics code number = IR.GUMS.REC.1401.174). Formal oral and written assent were obtained when the patients were informed of the study objectives.

### Measurements of anthropometry and clinical data collection

Measurements of obesity indices such as weight (kg), height (m), and BMI (kg/m^2^) were performed. To achieve precise measures, professional healthcare staff measured weight without shoes and with minimal clothing. A Seca 755 dial was used to determine weight via medical scale column (weighing accuracy of 0.5 kg). Height was measured to the nearest 0.1 cm via standard stadiometer. Height was measured without shoes and with shoulders in a neutral position. BMI was computed by dividing weight (kg) by height (m) squared. A BMI of ≥25 or ≥ 30 is considered overweight or obese, respectively. Prior to initiation of the study, all patient demographic and anthropometric data were collected. Past medical history was determined (including history of hypertension, T2DM, hyperlipidemia, and ever-smoking) as well as history of medications consumption and medications prescribed after angiography for patients with a CAD diagnosis: antihypertensive drugs [particularly beta blockers, thiazides, angiotensin II receptor blockers (ARBs); angiotensin-converting enzyme inhibitors (ACE inhibitors); calcium channel blockers (CCBs); antidiabetic medications (including metformin, and/or sulfonylureas); antihyperlipidemic medications (mainly statins); proton-pump inhibitors (PPIs); and antiplatelets including aspirin and clopidogrel (Plavix)].

### Angiography of the coronary arteries

Two cardiologists, based on the Judkin technique, used a femoral approach to perform coronary angiographs, and the severity of atherosclerosis was examined visually. Normal angiograms showed no apparent atherosclerotic changes in the coronary arteries, interpreted by cardiologists who were blinded to study details. The presence of stenosis in one, two, or three main coronary arteries was used as evidence of single-, two-, or three-vessel coronary artery disease. All patients with CAD underwent echocardiology in fewer than 3 days from hospitalization, in order to estimate left ventricular systolic ejection (LVEF). Based on the International Simpson method, two independent echocardiologists assessed the graphs again.

### Laboratory analyses

All patients were fasted for at least 8 h before 10 mL venous blood samples were drawn. To prevent coagulation, the samples were stored with sodium citrate in tubes at −20 degrees Celsius until the concentrations of fasting blood glucose (FBS) and total cholesterol were determined using the enzymatic-colorimetric method in accredited laboratories based on the manufacturer’s instructions (38). The enzymatic method (MAN CO., Tehran, Iran) was used to estimate high-density lipoprotein cholesterol (HDL-C) levels and serum uric acid levels, applying the uricase–peroxidase system (39). Triglyceride levels were measured using the enzymatic method through applying glycerol phosphate oxidase and using Bionic corporation commercial kits (MAN Co., Tehran, Iran). Themethod and Fried Wald formula was used in order to estimate low-density lipoprotein cholesterol (LDL-C) levels (40–42). Additionally, hemoglobin A1c (HbA1c) levels were measured and compared only among patients with a past medical history of T2DM (*n* = 399). As mentioned in the URRAH Study, total and cardiovascular mortality were predicted based on the level of SUA in diabetic patients using a 5.6 mg/dL cut point as a clinical margin (3). Thus, we considered those with SUA ≥ 5.6 mg/dL to have hyperuricemia.

### Novel insulin resistance surrogate markers

According to reported methods from previous studies, the insulin resistance surrogate indicators were calculated using the following formulas:


31 $$\begin{array}{l} \mathrm{Triglyceride glucose}\;\left(\mathrm{TyG}\right)\;\mathrm{index}=\\ \mathrm{Ln}\left(\frac{\mathrm{fasting triglycerides}\left(\frac{\mathrm{mg}}{\mathrm{dL}}\right)\times \mathrm{fasting glucose}\left(\frac{\mathrm{mg}}{\mathrm{dL}}\right)}{2}\right) \end{array}$$

31,35 $$\begin{array}{l} \mathrm{Triglyceride glucose}\left(\mathrm{TyG}\right)-\\ \;\;\;\;\;\;\;\;\;\;\;\;\;\;\;\mathrm{BMI}\mathrm{index}=\;\;\;\left(\mathrm{TyG}\mathrm{index}\times \mathrm{BMI}\right) \end{array}$$

### Statistical methods

No apriori statistical power calculations were performed. Hence, the current sample size (*n* = 2,000 non-CAD and CAD subjects) was determined according to our previous experience with this design. Due to the study methods, there were no missing data. Shapiro–Wilk tests were used to determine whether data were normally distributed. Categorical data were determined as frequency and %, and chi-squared or Fisher’s exact tests were used to analyze between-group differences. For continuous data, differences in the mean values were determined via independent samples *t*-tests and means and standard deviations (SD) were reported. The odds of hyperuricemia were examined for the novel markers under study using logistic regression. Biological sex, age, and history of hypertension, T2DM, hyperlipidemia, or using antihypertensive, antidiabetic, or anti-hyperlipidemic medications, in addition to ever- smoking status were all added as control variables for the fully adjusted model. By treating the median values of each tertile as continuous variables, we also tested for linear trends (*p*-values for trend) corresponding with odds of hyperuricemia across tertiles of insulin resistance surrogate markers, and odds ratios (OR) with corresponding 95% confidence intervals (CIs) were provided. Besides, Pearson correlation tests used for the analysis of correlation between serum uric acid and TyG index and TyG-BMI levels among CAD and non-CAD patients, and the correlation coefficient, 95% confidence intervals (CIs) and *p*-values are presented. IBM SPSS software was used for all analyses (version 24.0; SPSS, Chicago, IL).

## Results

### Baseline characteristics

Baseline demographic, anthropometric, and clinical characteristics of study participants, with and without CAD according to hyperuricemia status, are provided in Table 1. Overall, 1,170 patients were enrolled in the present cross-sectional study, with an average age of 52 (8) and 48 (8) years for patients with CAD (*n* = 814) and without CAD (*n* = 356), respectively. Participants were then divided into two groups on the basis of whether or not they had hyperuricemia (SUA levels above 5.6 mg/dL). Among the 814 patients with CAD, 355 had hyperuricemia, of whom 47.3% were male. Out of 356 patients without CAD, 92 had hyperuricemia, of whom 44.6% were male (Table 1). Patients with CAD who had hyperuricemia were more likely to be men and ever-smokers, had higher BMIs, and were more likely to have a history of hypertension, hyperlipidemia, and use of anti-hyperlipidemic medications (*p*-values < 0.05). Additionally, these patients had higher levels of SUA, triglycerides, TyG, and TyG-BMI, as compared to patients with CAD who did not have hyperuricemia (*p*-value < 0.001). However, there were no significant differences between the groups for total cholesterol or FBS levels (Table 1). Similarly, patients without CAD with hyperuricemia had significantly greater concentrations of SUA, triglycerides, TyG, and TyG-BMI than those without hyperuricemia, whereas patients in these subgroups were similar in terms of proportion of males/females, age, BMI, past medical histories, medication use, and other clinical and laboratory characteristics. Moreover, the HbA1c levels of diabetic patients, both with and without CAD, were not significantly different (Table 1).

**Table 1:** Baseline demographic, anthropometric, and clinical characteristics of study participants, with and without coronary artery disease (CAD) according to hyperuricemia status.

	Studied group						
	Non-CAD patients (*n* = 356)			CAD patients (*n* = 814)			
	Hyperuricemia status			Hyperuricemia status			
	SUA < 5.6 (*n* = 264)	SUA ≥ 5.6 (*n* = 92)	*P*-value	SUA < 5.6 (*n* = 459)	SUA ≥ 5.6 (*n* = 355)		*P*-value
Biological sex number of males (percentage)	97 (36.7%)	41 (44.6%)	0.214	169 (36.8%)	168 (47.3%)		0.003
Hypertension history number (percentage)	13 (4.9%)	9 (9.8%)	0.128	202 (44.0%)	182 (51.3%)		0.040
Using antihypertensive medications ^α^ (percentage)	11 (4.2%)	8 (8.7%)	0.109	185 (40.3%)	162 (45.6%)		0.134
T2DM history number (percentage)	30 (11.4%)	13 (14.1%)	0.464	203 (44.2%)	153 (43.1%)		0.776
Using antidiabetic medications ^β^ (percentage)	29 (11.0%)	13 (14.1%)	0.454	193 (42.0%)	141 (39.7%)		0.518
Hyperlipidemia history number (percentage)	21 (8.0%)	13 (14.1%)	0.099	112 (24.4%)	135 (38%)		<0.001
Using antihyperlipidemic medications ^γ^ (percentage)	20 (7.6%)	10 (10.9%)	0.383	97 (21.1%)	117 (33.0%)		<0.001
Ever- smokers	18 (6.8%)	6 (6.5%)	1.000	56 (12.2%)	92 (25.9%)		<0.001
Age, (years) (mean, SD)	49 (8)	48 (8)	0.507	53 (8)	52 (8)		0.757
BMI, kg/m^2^ (mean, SD)	28.05 (4.78)	29.02 (4.60)	0.093	27.99 (3.81)	29.04 (4.07)		<0.001
Serum biochemical analysis [mean (SD)]							
SUA (mg/dL)	3.0 (1.6)	6.5 (1.0)	<0.001	4.4 (0.7)	7.4 (2.1)		<0.001
Triglycerides (mmol/L)	1.51 (0.65)	1.67 (0.63)	0.043	1.69 (0.96)	1.98 (1.01)		<0.001
Total cholesterol (mmol/L)	4.57 (0.79)	4.51 (0.92)	0.532	4.59 (1.04)	4.64 (1.15)		0.533
Fasting blood sugar (mmol/L)	5.47 (1.45)	5.55 (1.44)	0.630	7.57 (3.78)		7.40 (3.53)	0.505
HbA1c (%) ^*^	6.27 (1.29)	6.68 (1.36)	0.359	6.54 (1.46)		6.47 (1.67)	0.690
Triglyceride glucose (TyG) index	8.66 (0.58)	8.80 (0.54)	0.049	9.01 (0.66)	9.18 (0.65)		<0.001
Triglyceride glucose (TyG)-BMI	243.62 (47.40)	255.64 (44.8)	0.034	252.23 (38.45)	266.76 (44.35)		<0.001

CAD, coronary artery disease; BMI, body mass index; SUA, serum uric acid; SD, standard deviation.

^α^ Mainly include beta blockers, thiazides, ARBs (Angiotensin II receptor blockers), ACE inhibitors (Angiotensin-converting enzyme inhibitors), and CCBs (calcium channel blockers).

^β^ Mainly include metformin, and/or sulfonylureas.

^γ^ Mainly include statins.

^*^ Measured and compared only among patients with a history of T2DM (*n* = 399).

CAD patients’ clinical characteristics, including categorization of CAD types (the numbers of involved vessels based on angiographic findings), LVEF, and prescribed medications according to hyperuricemia status, are provided in Table 2. About one-third of CAD patients without hyperuricemia had nonobstructive CAD (32.2%), while a greater proportion of those with hyperuricemia were diagnosed with three-vessel coronary disease (33.2%). Those with hyperuricemia also tended to have lower LVEF. All CAD patients were prescribed antiplatelets, statins, PPIs, and antihypertensive medication, regardless of hyperuricemia status (Table 2).

**Table 2:** Clinical characteristics of patients with CAD according to hyperuricemia status.

	CAD patients (*n* = 814)		Hyperuricemia status		SUA < 5.6 mg/dL (*n* = 459) Cases (%)	SUA ≥ 5.6 mg/dL (*n* = 355) Cases (%)
Nonobstructive CAD	148 (32.2%)	96 (27.0%)
One-vessel coronary disease	91 (19.8%)	80 (22.5%)
Two-vessel coronary disease	81 (17.6%)	61 (17.2%)
Three-vessel coronary disease	139 (30.3%)	118 (33.2%)
LVEF mean (SD)	47 (10)	44 (12)
Prescribed medications after angiography		
Antiplatelets ^α^ (number, percentage)	459 (100%)	355 (100%)
Statins ^β^ (number, percentage)	459 (100%)	355 (100%)
Antihypertensive ^γ^ (number, percentage)	459 (100%)	355 (100%)
PPIs ^£^ (number, percentage)	459 (100%)	355 (100%)

CAD, coronary artery disease. LVEF, left ventricular ejection fraction. PPIs, proton-pump inhibitors.

^α^ Mainly include aspirin, clopidogrel (Plavix).

^β^ Mainly include atorvastatin.

^γ^ Mainly include ARBs (Angiotensin II receptor blockers), ACE inhibitors (Angiotensin-converting enzyme inhibitors), CCBs (calcium channel blockers).

^£^ Mainly include pantoprazole.

**Figure 2:**
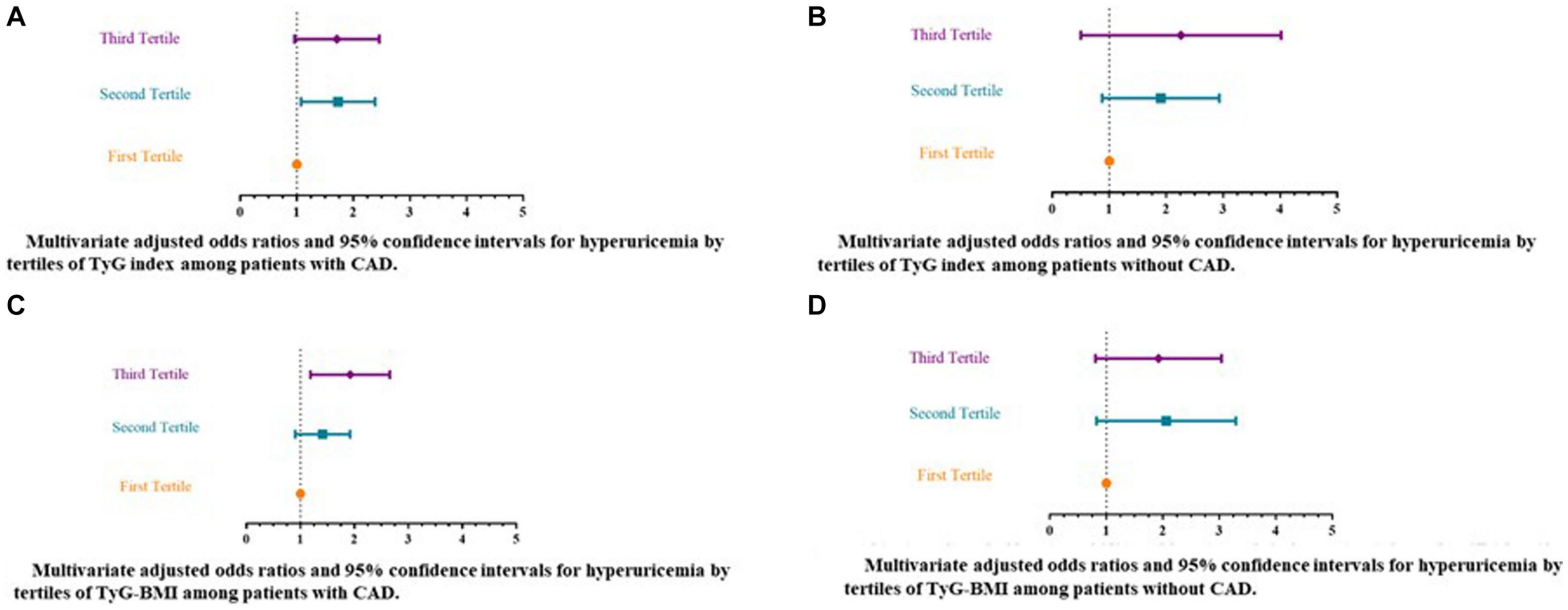
Multivariate adjusted odds ratios and 95% confidence intervals for hyperuricemia by tertiles of insulin resistance surrogate markers [triglyceride-glucose (TyG) index and triglyceride-glucose-body mass index (TyG-BMI)]. **(A)** Multivariate adjusted odds ratios and 95% confidence intervals for hyperuricemia by tertiles of TyG index among patients with CAD. **(B)** Multivariate adjusted odds ratios and 95% confidence intervals for hyperuricemia by tertiles of TyG index among patients without CAD. **(C)** Multivariate adjusted odds ratios and 95% confidence intervals for hyperuricemia by tertiles of TyG-BMI among patients with CAD. **(D)** Multivariate adjusted odds ratios and 95% confidence intervals for hyperuricemia by tertiles of TyG-BMI among patients without CAD. ^a^ Multiple regression model adjusted for biological sex; age, and history of hypertension, T2DM, or hyperlipidemia, using antihypertensive ^α^, antidiabetic ^β^, or antihyperlipidemic medications ^γ^, and ever-smoking status. ^α^ Mainly include Beta blockers, Thiazides, ARBs (Angiotensin II receptor blockers), ACE inhibitors (Angiotensin-converting enzyme inhibitors), and CCBs (calcium channel blockers). ^β^ Mainly include metformin, and/or sulfonylureas. ^γ^ Mainly include statins. TyG, triglyceride-glucose index. CAD, Coronary artery disease. TyG-BMI, triglyceride-glucose-body mass index.

Table 3 and Figures 2A–D present the unadjusted and adjusted ORs and associated 95%CIs for hyperuricemia according to the tertiles of insulin resistance surrogate indicators, in patients with and without CAD. According to the crude regression models, when exploring the relationship between TyG and odds of hyperuricemia among those with CAD, compared to patients in the 1st tertile of TyG (median = 8.37), the patients in both the 2nd (median = 8.95) (OR = 1.62, 95% CI 1.128–2.34) and 3rd tertiles (median = 9.62) (OR = 1.85, 95% CI 1.30–2.63) had significantly higher odds of hyperuricemia (*p*-value for trend = 0.001). Likewise, after controlling for potential confounders including age, biological sex, history of T2DM, hyperlipidemia, and hypertension, using antihypertensive, antidiabetic, or antihyperlipidemic medications, and ever-smoking status in the multiple regression models, it was found that those in the 2nd and 3rd tertiles of TyG had 60–65% higher odds of hyperuricemia (OR = 1.65, 95% CI 1.12–2.42; and OR = 1.60, 95% CI 1.02–2.51, respectively) compared with patients in the 1st tertile (*p*-value for trend = 0.036) (Table 3; Figure 2A). In the patients without CAD, although an overall significant association between TyG and hyperuricemia was detected (*p*-value for trend = 0.043) in the unadjusted model, only those in the 2nd tertile of TyG (median = 8.95) had elevated odds of hyperuricemia (OR = 1.81, 95% CI 1.05–3.10). Nonetheless, after multivariable model adjustment for relevant confounders, no significant associations remained (*p*-value for trend = 0.072) (Table 3; Figure 2B).

**Table 3:** Odds ratios and 95% confidence intervals for hyperuricemia according to tertiles of insulin resistance surrogate markers.

	Tertiles of insulin resistance surrogate markers			*P*-value^d^
	1st tertile	2nd tertile	3rd tertile	
Triglyceride glucose (TyG) index				
CAD patients				
Cases/non-cases	76/148	122/146	157/165	
Median	8.37	8.95	9.62	
Crude model	1.00	1.62 (1.12–2.34)	1.85 (1.30–2.63)	0.001
Multivariable adjusted model ^a^	1.00	1.65 (1.12–2.42)	1.60 (1.02–2.51)	0.036
Non-CAD patients				
Cases/non-cases	33/132	38/84	21/48	
Median	8.23	8.95	9.38	
Unadjusted model	1.00	1.81 (1.05–3.10)	1.75 (0.92–3.31)	0.043
Multivariable adjusted model ^a^	1.00	1.72 (0.98–3.01)	1.81 (0.77–4.20)	0.072
Triglyceride glucose (TyG)-BMI				
CAD patients				
Cases/non-cases	86/158	129/171	140/130	
Median	217.61	251.90	296.45	
Crude model	1.00	1.38 (0.97–1.96)	1.97 (1.38–2.82)	<0.001
Multivariable adjusted model ^a^	1.00	1.35 (0.94–1.95)	1.83 (1.24–2.70)	0.002
Non-CAD patients				
Cases/non-cases	29/116	28/63	35/85	
Median	200.81	253.82	294.84	
Crude model	1.00	1.77 (0.97–3.24)	1.64 (0.93–2.90)	0.088
Multivariable adjusted model ^a^	1.00	1.81 (0.97–3.40)	1.71 (0.93–3.13)	0.087

CAD, coronary artery disease; SUA, serum uric acid; BMI, body mass index.

^a^ Multiple regression model adjusted for biological sex; age, and history of hypertension, T2DM, or hyperlipidemia, using antihypertensive ^α^, antidiabetic ^β^, or antihyperlipidemic medications ^γ^, and ever-smoking status.

^α^ Mainly include beta blockers, thiazides, ARBs (Angiotensin II receptor blockers), ACE inhibitors (Angiotensin-converting enzyme inhibitors), and CCBs (calcium channel blockers).

^β^ Mainly include metformin, and/or sulfonylureas.

^γ^ Mainly include statins.

^d^ *p*-value less than 0.05 considered as statistical significant.

With respect to the relationship between TyG-BMI index and hyperuricemia among the studied groups, according to the crude regression models, significant increases in odds of hyperuricemia were noted among patients with CAD in the 3rd tertile of the TyG-BMI index (median = 296.45) (OR = 1.97, 95% CI 1.38–2.82) as compared with those in the 1st tertile (median = 217.61) (*p*-value for trend < 0.001). This elevated odds of hyperuricemia remained significant following adjustment for confounding factors and was approximately 83% for CAD patients in the highest tertile of TyG-BMI as compared to the lowest (OR = 1.83, 95% CI 1.24–2.70; *p*-value for trend = 0.002) (Table 3; Figure 2C). However, no significant relationships between TyG-BMI index and hyperuricemia were indicated among patients without CAD in either unadjusted or multiple regression models (Table 3; Figure 2D).

**Figure 3:**
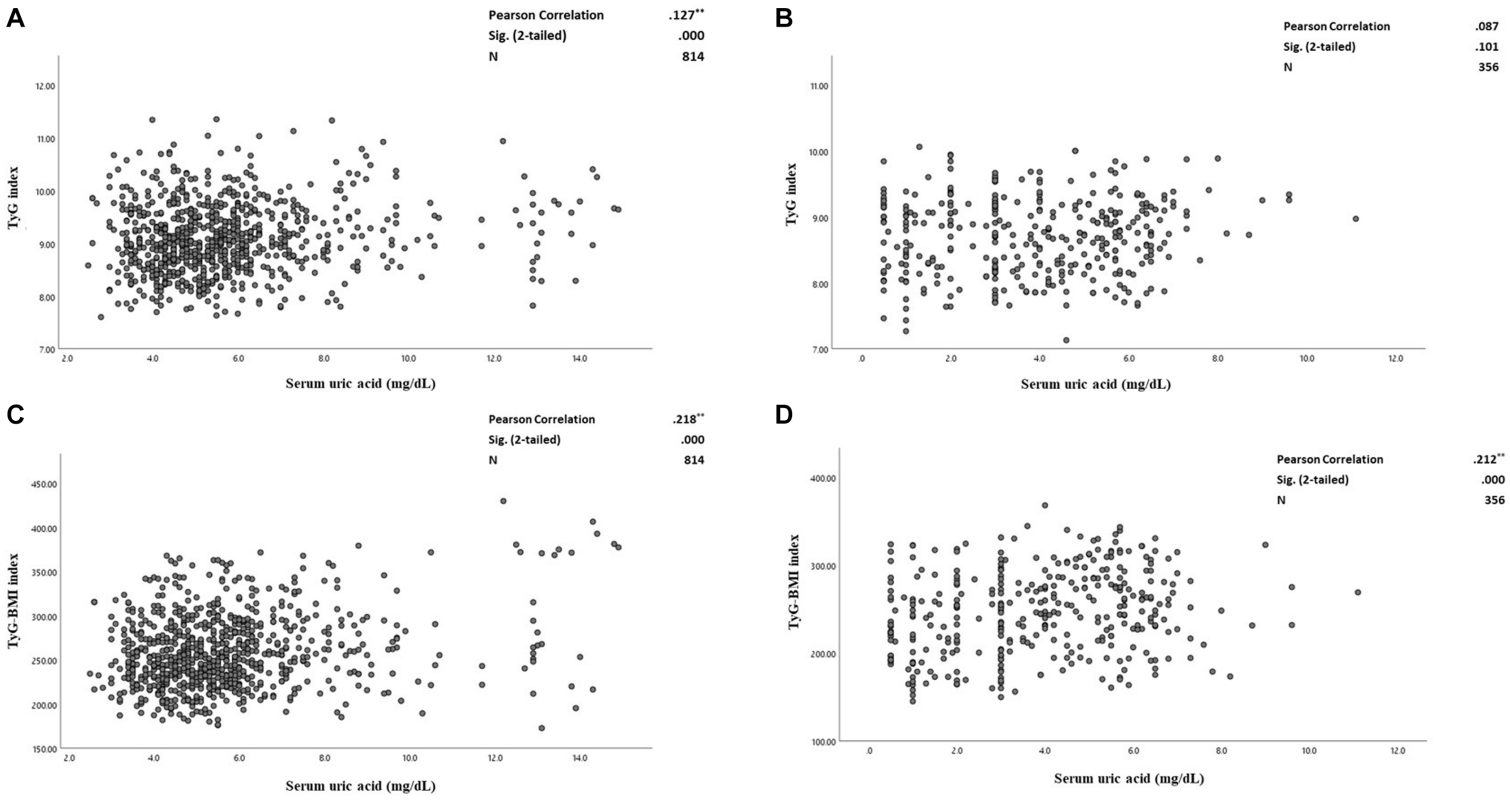
**(A–D)** The correlation between serum uric acid and triglyceride-glucose (TyG) index and triglyceride-glucose-Body mass index (TyG-BMI) levels among CAD and non-CAD patients. **(A)** The correlation between serum uric acid and TyG index among the patients with CAD. **(B)** The correlation between serum uric acid levels and TyG index among the patients without CAD. **(C)** The correlation between serum uric acid and TyG-BMI indices among the patients with CAD. **(D)** The correlation between serum uric acid levels and TyG-BMI indices among the patients without CAD. Pearson correlation tests used and the correlation coefficient, 95% confidence intervals (CIs) and *p*-values are presented. TyG, triglyceride-glucose index. CAD, Coronary artery disease. TyG-BMI, triglyceride-glucose-body mass index.

Figures 3A–D plots SUA levels by insulin resistance surrogate markers (TyG and TyG-BMI indices) among patients with and without CAD. The correlation between TyG and SUA levels was not significant among the patients without CAD. Whereas, among patients with CAD, there was a weak positive correlation between TyG and SUA levels (*r* = 0.127; *p*-value < 0.001) (Figures 3A,B). Figures 3C,D plot SUA levels against TyG-BMI in the two studied groups. There were significant weak positive correlations between TyG-BMI and SUA concentrations among both patients with CAD (*r* = 0.218; *p*-value < 0.001) and without CAD (*r* = 0.212; *p*-value < 0.001).

## Discussion

The primary purpose of the current study was to examine the associations between insulin resistance status as assessed by novel surrogate markers including TyG and TyG-BMI, and odds of hyperuricemia among patients with and without CAD. Results indicated that these novel indices could predict risk of hyperuricemia among CAD patients, regardless of potential confounders. Although neither the unadjusted (except for TyG index), nor the multiple regression models revealed statistically significant relationships in patients without CAD, there were significant weak positive correlations between TyG-BMI and SUA concentration in both the CAD and non-CAD groups. However, high TyG index showed a weak positive association with high levels of SUA only among the CAD group.

Physiological concentration of SUA, the metabolic byproduct of purine nucleotides, has beneficial powerful antioxidant effects and works as a free radical scavenger. Moreover, the protective effects of SUA include DNA damage resistance, anti-osteoporotic action, and postponement of cognitive decline. With excessive concentrations, high uric acid levels or hyperuricemia followed by aggregated production of reactive oxygen species (ROS) under conditions of stress, such as hypoxia and ischemia would occur (8). Ongoing epidemiological studies suggest that there are global increases in circulating levels of SUA (43). Hence, a definitive threshold level for diagnosis of hyperuricemia associated with increased risk of chronic metabolic disorders, that might also be considered as an important routine clinical test for patients with such disorders, could be clinically beneficial (44). Results from the URRAH research indicated that regardless of age, gender, the history of T2DM or hypertension, patients with metabolic syndrome had a greater risk of cardiovascular death when they had hyperuricemia, as assessed by a cut-off level of 5.6 mg/dL (3). Additionally, one cohort study conducted in China, showed that increased SUA was considered as a risk factor for CAD, regardless of obesity status (45). The same results were obtained in a cohort study conducted in the United States, where a positive correlation between elevated SUA levels and CVD disease risk factors was shown (46). Recently, some research has studied the correlations between hyperuricemia or elevated SUA levels and not only CVD, but also several metabolic disorders such as T2DM, kidney disease, and hypertension (20, 22, 47–49).

With respect to this evidence, in the current cross-sectional study, we aimed to explore two novel insulin resistance surrogate markers (TyG and TyG-BMI) as predictors for odds of hyperuricemia among patients with and without CAD. TyG index, as a promising surrogate measure for insulin resistance in large-scale epidemiological investigations given its simplicity of use and affordability, had a strong correlation with TyG-BMI, TyG-waist circumference (TyG-WC), HOMA-IR, and HbA1c (50). This novel biomarker has been considered as an effective marker in diagnosis of some chronic diseases such as metabolic syndrome and T2DM (51). Interestingly, a recently published study reported the higher the TyG index value, the higher the risk of CVDs over a 10-year period (29). Moreover, the inclusion of this index in the Framingham risk score (FRS) showed an added value, enhancing the predictive power of this score for CVD risk evaluation (52).

Similarly, TyG-BMI, which measures the TyG index multiplied by the BMI, is thought to be another reliable marker for diagnosing insulin resistance as compared with conventional lipid measures including blood lipid ratios, blood glucose markers, and obesity-related parameters. Additionally, TyG-BMI has been linked to prehypertension, nonalcoholic fatty liver disease, and stroke in a number of recent investigations (53–55).

The present research outcomes indicate positive correlations between TyG and SUA only among patients with CAD, and between TyG-BMI and SUA in patients both with and without CAD, though correlations were weak, further supporting the associations between higher insulin resistance and hyperuricemia particularly among patients suffering from CAD. However, it should be noted that there might be some possibility of spurious correlations, or statistically significant results found by random chance as the observed correlation coefficients between the studied parameters were not very strong. Regardless of this limitation, these findings expand the currently available evidence indicating that elevated levels of serum lipids and blood glucose, as indicated in insulin resistance alternative markers particularly TyG-BMI, were significantly associated with the odds of hyperuricemia (7, 8, 56–62). Shi et al. (58), conducted a case–control study on hyperurecemic patients (*n* = 339) compared to control subjects (*n* = 6,127), aiming to explore the utility of the TyG index for estimating hyperuricemia risk among a Chinese population. In parallel with the current study, results indicated that subjects in the fourth quartile of TyG index had a significant increased risk for hyperuricemia than those in the first quartile. More importantly, results indicated that simultaneous lipid and glycemic control is necessary for hyperuricemia prevention (58). Align with these findings, Kahaer et al. investigated the relationship between a number of obesity-related risk factors and risk of hyperuricemia (defined as an SUA level > 7.0 mg/dL) among a total of 2,243 Chinese subjects, an even more robust association was revealed when exploring the TyG index in relation to hyperuricemia risk (56). Additional reports have indicated that the TyG index could significantly predict the risk of hyperuricemia among subjects diagnosed with hypertension (7, 57, 60). For example, in a cross-sectional study among Chinese older adults in which TyG and TyG-BMI indices were used as insulin resistance biomarkers, these markers were shown to be significantly associated with increased risk of hyperuricemia or hypertension alone or in combination (60). Interestingly, such relationships seem to be consistent in patients with T2DM (8, 20, 62). In a cross-sectional study conducted by Hang et al., it was reported that in patients with T2DM, a weak but significant correlation was detected between SUA and insulin resistance as indicated by TyG (*r* = 0.406, *p*-value < 0.05) and the triglyceride to HDL-C ratio (*r* = 0.493, *p*-value < 0.05). Additionally, TyG-BMI (*r* = 0.272, *p*-value < 0.05) and METS-IR (*r* = 0.238, *p*-value < 0.05) showed very weak, but still significant correlations with insulin resistance (8). These results were consistent with another study showing a significant association between elevated SUA and higher risk of insulin resistance, especially in women with T2DM (20). A recently published study by Qi et al., additionally confirmed that TyG could be considered as a risk factor for developing hyperuricemia among patients suffering from nonalcoholic fatty liver disease (NAFLD), regardless of potential confounders (63). Of note, another recent study suggested that either of the factors, hyperuricemia or elevated TyG index, could independently predict the risk of major adverse cardiovascular event (MACE) occurrence among those who underwent coronary artery bypass grafting (CABG). Intriguingly, the two predictivefactorsshowed a synergistic interaction. As such, the highest risk of reporting MACE was noted among the subjects presenting with increased TyG index and serum uric acid levels simultaneously, as compared to those with lower levels of either of these factors (64). These reports confirm the added value of considering both insulin resistance and hyperuricemia indicators when assessing risk for CVDs and comorbid complications.

Since a causal relationship between insulin resistance and SUA has not been determined, a definite mechanism underlying the association between these two conditions cannot be confirmed. Notably, one of the primary predictors of insulin resistance is believed to be excess adipose tissue, which then could be involved in inducing oxidative stress (65). Additionally, insulin resistance promotes SUA synthesis via the hexose monophosphate route, while decreasing SUA renal excretion (66, 67). One more important point is that insulin escalates renal reabsorption of uric acid via stimulation of glucose transporters (Glut 9) (encoded by SLC2A9) and other renal urate transporters participate in reabsorption of uric acid (10). In other words, insulin resistance and resultant hyperinsulinemia encourage the renal tubules to reabsorb uric acid. Accordingly, this condition could then enhance the creation of fat cells in the liver, leading to aberrant purine metabolism and a subsequent increase in SUA levels (68). However, some studies have also suggested that central adiposity and SUA accumulation may contribute to insulin resistance (67). As mentioned, augmented SUA levels may lead to oxidative stress, which then may impair glucose metabolism and reduce insulin sensitivity, and might contribute to insulin resistance by upregulating insulin receptor substrate 1 phosphorylation, as well as increased production of excessive ROS (66–68). Additionally, the ROS formation escalation following hyperuricemia could result in a decrease in the transcription factors necessary for the expression of the insulin gene, and a reduction in insulin synthesis and release (69).

### The clinical importance of the current findings

Despite the fact that the cross-sectional design of the current study limits the ability to determine causal relationships between the studied exposures and outcomes, as well as their clinical interpretation, the present study suggested that TyG and TyG-BMI indices, along with SUA levels may serve as feasible and practical clinical assessments to determine risk for hyperuricemia and atherosclerotic-related conditions. Accordingly, it seems that employing these available and inexpensive measures could assist in risk stratification and early detection of CAD patients who may require more comprehensive treatment options in association with insulin resistance. Nonetheless, long-term prospective cohort investigations are necessary to confirm the diagnostic value of these novel indices for early detection of insulin resistance and hyperuricemia, as well as associated CAD comorbidities. Future studies should also consider establishing cut points for optimal balance between positive and negative-predictive values of the surrogate markers of insulin resistance.

### Study strengths and limitations

Several strengths are attributed to the current study. Foremost, this is the first study to assess the odds of hyperuricemia based on novel insulin resistance surrogate markers among patients with and without CAD in a sample of the Iranian population. Another strength is that CAD diagnosis was established using findings from angiograms, interpreted by two expert interventional cardiologists. Consequently, miscategorization of CAD cases was minimized.

The present research study has also limitations which should be considered when interpreting findings. First, the cross-sectional, retrospective, and single-center properties of the study limit interpretation as well as generalizability. Associations between the novel insulin resistance indices and incidence of future hyperuricemia events, gout, and related morbidity and mortality could not be explored because of absence of follow-up data. Additionally, due to absence of data on a number of relevant explanatory variables including waist circumference, dietary intakes, sleeping habits, and physical activity, the potential effects of these variables on the study biomarkers and outcomes could not be considered.

## Conclusion

The current cross-sectional study of 814 angiographically-confirmed patients with CAD and 356 patients without CAD, revealed that two novel insulin resistance surrogate markers, TyG and TyG-BMI indices, were associated with increased odds of hyperuricemia, regardless of potential confounders. For patients with CAD, those in the highest tertiles for both TyG and TyG-BMI, had increased odds for hyperuricemia by 60 and 83%, respectively. Although no significant relationships were indicated for patients without CAD, significant positive and weak correlations between TyG-BMI and SUA concentrations among patients with and without CAD were noted. However, the correlation between elevated TyG index scores, and elevated levels of SUA appeared to be significant only among patients with CAD.

All together, the present study findings indicate that stratifying the patients based on their SUA levels and insulin resistance surrogate indices may facilitate primary screening and early diagnosis of CAD patients who may require more comprehensive treatment options associated with insulin resistance. However, further research with stronger study designs is needed to confirm these outcomes.

## Data availability statement

The raw data supporting the conclusions of this article will be made available by the authors, without undue reservation.

## Ethics statement

The study protocol was assessed and approved by the Cardiovascular Diseases Research Center Institutional Review Board [affiliated with Guilan University of Medical Sciences (GUMS)] (registered with research number = 4246). The GUMS’ Ethics Committee also approved this study (ethics code number = IR.GUMS.REC.1401.174). The studies were conducted in accordance with the local legislation and institutional requirements. The participants provided their written informed consent to participate in this study.

## Author contributions

MM-R and ZG conceived and designed the research. MM-R, ZG, SA, and AH played an important role in data collection and reviewing the patients’ documents. MM-R, ZG, and NS acquired data and performed data analysis. MM-R, ZG, SM, NS, and SR played an important role in results interpretation and drafted and revised the manuscript. All authors reviewed and approved the final version of the submitted manuscript.

## Conflict of interest

The authors declare that the research was conducted in the absence of any commercial or financial relationships that could be construed as a potential conflict of interest.

## Publisher’s note

All claims expressed in this article are solely those of the authors and do not necessarily represent those of their affiliated organizations, or those of the publisher, the editors and the reviewers. Any product that may be evaluated in this article, or claim that may be made by its manufacturer, is not guaranteed or endorsed by the publisher.
